# Staging and quantification of florbetaben PET images using machine learning: impact of predicted regional cortical tracer uptake and amyloid stage on clinical outcomes

**DOI:** 10.1007/s00259-019-04663-3

**Published:** 2019-12-28

**Authors:** Jun Pyo Kim, Jeonghun Kim, Yeshin Kim, Seung Hwan Moon, Yu Hyun Park, Sole Yoo, Hyemin Jang, Hee Jin Kim, Duk L. Na, Sang Won Seo, Joon-Kyung Seong

**Affiliations:** 1 Department of Neurology, Samsung Medical Center, Seoul, South Korea; 2 Samsung Alzheimer Research Center, Samsung Medical Center, Seoul, South Korea; 3 Neuroscience Center, Samsung Medical Center, Seoul, South Korea; 4 Department of Bio-convergence Engineering, Korea University, Seoul, South Korea; 5 Department of Neurology, Kangwon National University Hospital, Chuncheon, South Korea; 6 Department of Nuclear Medicine, Samsung Medical Center, Seoul, South Korea; 7 Department of Cognitive Science, Yonsei University, Seoul, South Korea; 8 Department of Health Sciences and Technology, SAIHST, Sungkyunkwan University, Seoul, South Korea; 9 Department of Clinical Research Design & Evaluation, SAIHST, Sungkyunkwan University, Seoul, South Korea; 10 Center for Clinical Epidemiology, Samsung Medical Center, Seoul, South Korea; 11 School of Biomedical Engineering, Korea University, Seoul, South Korea; 12 Department of Artificial Intelligence, Korea University, Seoul, South Korea

**Keywords:** Amyloid PET, Staging, Quantification, Alzheimer’s disease, Machine learning

## Abstract

**Purpose:**

We developed a machine learning–based classifier for in vivo amyloid positron emission tomography (PET) staging, quantified cortical uptake of the PET tracer by using a machine learning method, and investigated the impact of these amyloid PET parameters on clinical and structural outcomes.

**Methods:**

A total of 337 ^18^F-florbetaben PET scans obtained at Samsung Medical Center were assessed. We defined a feature vector representing the change in PET tracer uptake from grey to white matter. Using support vector machine (SVM) regression and SVM classification, we quantified the cortical uptake as predicted regional cortical tracer uptake (pRCTU) and categorised the scans as positive and negative. Positive scans were further classified into two stages according to the striatal uptake. We compared outcome parameters among stages and further assessed the association between the pRCTU and outcome variables. Finally, we performed path analysis to determine mediation effects between PET variables.

**Results:**

The classification accuracy was 97.3% for cortical amyloid positivity and 91.1% for striatal positivity. The left frontal and precuneus/posterior cingulate regions, as well as the anterior portion of the striatum, were important in determination of stages. The clinical scores and magnetic resonance imaging parameters showed negative associations with PET stage. However, except for the hippocampal volume, most outcomes were associated with the stage through the complete mediation effect of pRCTU.

**Conclusion:**

Using a machine learning algorithm, we achieved high accuracy for in vivo amyloid PET staging. The in vivo amyloid stage was associated with cognitive function and cerebral atrophy mostly through the mediation effect of cortical amyloid.

**Electronic supplementary material:**

The online version of this article (10.1007/s00259-019-04663-3) contains supplementary material, which is available to authorized users.

## Introduction

Amyloid positron emission tomography (PET) is a well-established and widely used method for biomarker-supported diagnosis of Alzheimer’s disease (AD). Assessments of amyloid positivity are often required for both clinical and research purposes, and in general, we use one of the two methods: visual assessment (VA) or automatic quantification. For VA, tracer uptake in the grey matter and the neighbouring white matter is compared. Although VA of amyloid PET scans shows high agreement with autopsy findings [1], since VA relies on the expertise of nuclear medicine physicians or neurologists, human errors or interrater discrepancy may occur depending on the rater’s experience.

Another method of assessing amyloid PET scans is the standardised uptake value ratio (SUVr)–based quantification method. SUVr is the ratio of the mean uptake value within the target region to that in the reference region. While the SUVr-based quantification method could be more objective than VA, there are some inherent limitations imposed by the need for a reference region. Generally, the cerebellum is used as the reference region since it shows scarce involvement of amyloid pathology. However, amyloid deposition in the cerebellum occurs in advanced AD, affecting the SUVr values [2]. In addition, an optimal cutoff value is required to determine whether PET scans show positive or negative amyloid deposition, and the normative data required to determine the cutoff value are not available in most centres.

In this regard, there has been some effort to develop machine learning algorithms to determine amyloid positivity. Previous studies have shown that automated classifiers using several different methods achieved excellent agreement with VAs [3, 4]. However, these classifiers assessed amyloid deposition in terms of presence or absence of amyloid. Pathological studies have shown that amyloid deposition in the striatum predicts a greater prevalence of dementia and clnicopathological AD [5, 6]. Indeed, several recent studies using amyloid PET have shown that striatal amyloid deposition is related to worse cognitive function and more rapid decline in cognitive function [7, 8]. Therefore, from a clinical standpoint, it would be necessary to determine whether amyloid deposits are present in the striatum. On the other hand, a pathological study has shown that the cortical amyloid burden increases with the advancement of the amyloid phase even after the involvement of subcortical structures [9]. Considering this result, there is a possibility that a concurrent increase in the cortical amyloid burden might have mediated the detrimental effects of striatal amyloid involvement on worse clinical outcomes. However, there is a lack of evidence clarifying whether striatal involvement or the cortical burden of amyloid has a more crucial impact on clinical outcomes.

In the present study, we developed a reference-free machine learning–based classifier that not only can determine amyloid positivity but also can detect striatal tracer uptake, allowing the determination of in vivo amyloid PET stages. We also defined a quantification variable that can be obtained from the same machine learning pipeline. While SUVr is based on the summation of uptake values, we quantified PET images based on the similarity of the uptake pattern to that of amyloid-positive scans. Finally, we assessed the clinical impact of both the in vivo amyloid stage and the cortical amyloid burden, and examined the mediation effect between the two PET parameters. We hypothesised that a higher cortical amyloid burden would be associated with worse cognitive function and more pronounced brain atrophy, possibly mediating the relationship between the negative effects of higher stage and clinical outcomes.

## Methods

### Participants

A total of 371 subjects (54 cognitively normal [CN] subjects, 155 patients with amnestic mild cognitive impairment [MCI], and 162 patients with AD dementia) who underwent florbetaben PET scans between August 2015 and April 2017 were recruited from the in-house PET registry of Samsung Medical Center (Seoul, Korea). AD dementia was diagnosed on the basis of National Institute on Aging–Alzheimer’s Association research criteria for probable AD [10]. Amnestic MCI was diagnosed on the basis of the Petersen criteria [11] and the presence of objective memory impairment of less than the 16th percentile of the norm in at least one memory test. CN subjects were all characterised by the absence of a history of neurologic or psychiatric disorders and normal cognitive function determined using neuropsychological tests (greater than the 16th percentile of the norm). All subjects were evaluated by comprehensive interviews, neurological examinations, and neuropsychological assessments. Blood tests to exclude secondary causes of dementia included a complete blood count, blood chemistry tests, vitamin B_12_/folate levels, syphilis serological tests, and thyroid function tests. Conventional brain MRI scans confirmed the absence of structural lesions such as tumours, traumatic brain injuries, hydrocephalus, or severe white matter hyperintensities.

### Ethics statement

The institutional review board at Samsung Medical Center approved this study, and informed consent was obtained from the patients and caregivers.

### PET image acquisition and analysis

Patients underwent 18F-florbetaben PET at Samsung Medical Center using a Discovery STe PET/CT scanner (GE Medical Systems, Milwaukee, WI, USA) in a three-dimensional scanning mode that examined 47 slices of 3.3-mm thickness spanning the entire brain. CT images were acquired using a 16-slice helical CT (140 KeV, 80 mA; 3.75-mm section width) for attenuation correction. A 20-min emission PET scan in the dynamic mode (consisting of 4 × 5 min frames) was performed 90 min after injection of 381-MBq 18F-florbetaben.

For VA, two raters (a nuclear medicine physician and a neurologist) assessed the florbetaben (^18^F) PET images according to a predefined regional cortical tracer uptake (RCTU) scoring system (1 = no binding, 2 = minor binding, 3 = pronounced binding) for four brain regions (frontal cortex, posterior cingulate cortex/precuneus, lateral temporal cortex, and parietal cortex) in each hemisphere [12]. In addition to these four brain regions, the presence of florbetaben uptake in the striatal region was rated using the methodology employed for VA of 18F-flutemetamol PET images, another amyloid PET technique that uses an 18F-labelled tracer [13]. Discrepancies between the assessments of the raters were resolved by consensus. The RCTU scores from the original four regions were then condensed into a single predefined three-grade scoring system for each PET scan, yielding the brain amyloid-β plaque load (BAPL) score (1 = no amyloid-β load, 2 = minor amyloid-β load, 3 = significant amyloid-β load) [12]. We defined 18F-florbetaben PET scans as positive when the VA score was 2 or 3 on the BAPL scoring system. In this VA step, 23 subjects were excluded due to poor-quality PET scans.

While the visually assessed amyloid stages were the primary target to predict with our classification model, we tested whether our method also works for the SUVr cutoff-based amyloid stage labels. To determine the subjects’ SUVr cutoff-based amyloid stages, the optimal cutoff values for cortical (1.104) and striatal (1.097) regions were applied. These cutoff values were derived by applying the iterative outlier method used by Mormino et al. [14] to cognitively normal subjects. For calculation of the SUVr values, we used the whole cerebellum as a reference region.

### MR image acquisition

All subjects underwent a 3D volumetric brain MRI scan. An Achieva 3.0-Tesla MRI scanner (Philips, Best, the Netherlands) was used to acquire 3D T1 Turbo Field Echo (TFE) MRI data using the following imaging parameters: sagittal slice thickness, 1.0 mm with 50% overlap; no gap; repetition time, 9.9 ms; echo time, 4.6 ms; flip angle, 8°; and matrix size, 240 × 240 pixels reconstructed to 480 × 480 over a field view of 240 mm.

### Image preprocessing

**Fig. 1:**
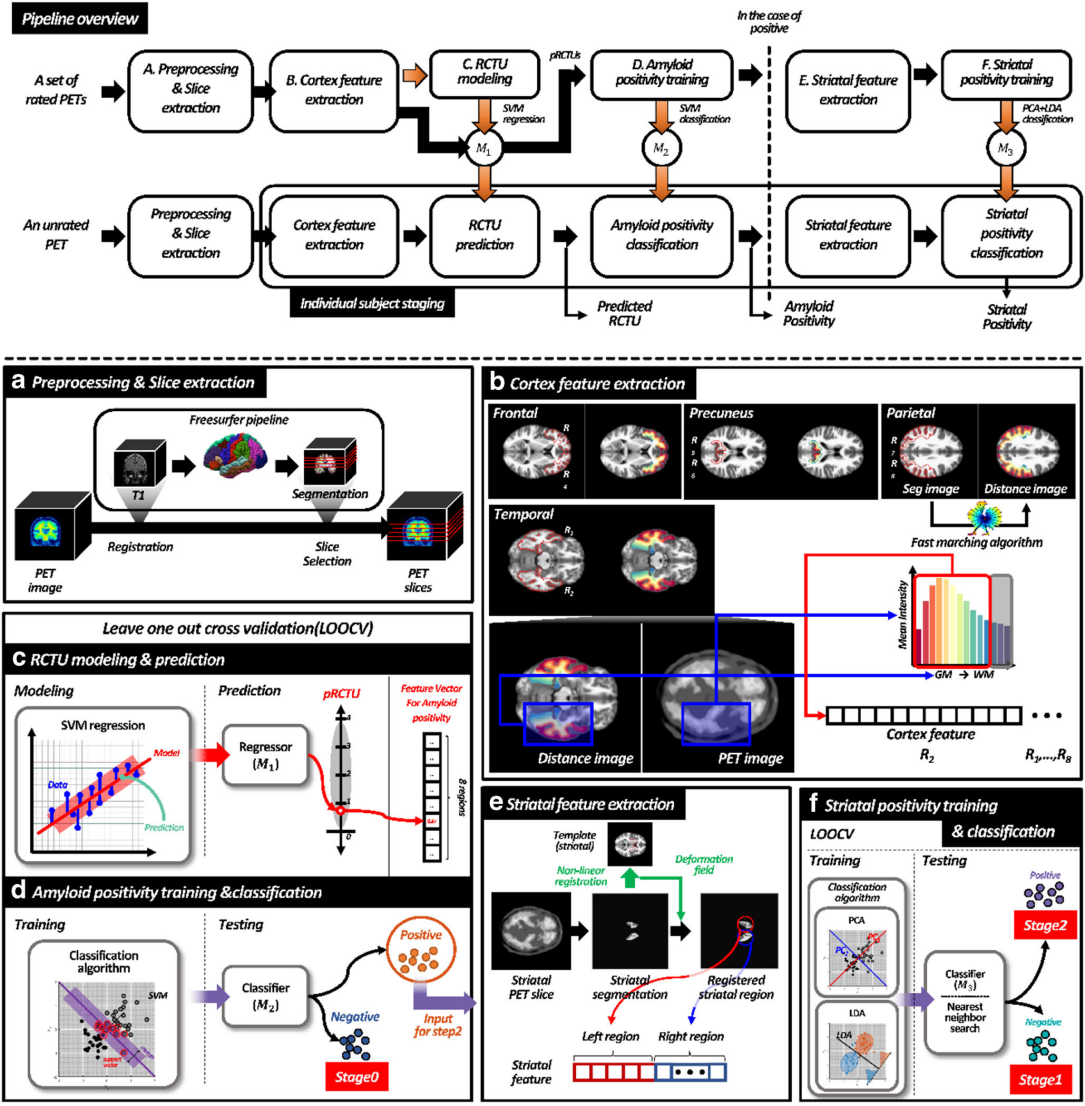
Overview of the proposed amyloid staging pipeline. (a) Image preprocessing and slice extraction. (b) Regional feature extraction for the cortex. (c) pRCTU modelling and prediction. (d) First step of staging: amyloid positivity classification. (e) Striatal feature extraction based on caudate and putaminal regions. (f) Second step of staging: striatal positivity classification. PET positron emission tomography, RCTU regional cortical tracer uptake, PCA principal component analysis, LDA linear discriminant analysis, LOOCV leave-one-out cross-validation

For automatic slice selection similar to that performed by human raters, we first segmented the T1-weighted MR image using FreeSurfer (version 5.1). The FreeSurfer is a suite of tools serving as a pipeline for automated surface model-based segmentation of volume images, of which each step is described online (http://surfer.nmr.mgh.harvard.edu/). Figure 1 a shows this process. First, skull-stripping was performed on the T1-weighted image. This step consists of motion correction, space transformation, and normalisation. Second, the image was segmented into grey and white matter, and the cortical surface was constructed using the segmented image. Next, individual surface registration was performed using spherical-based mapping and optimisation, which corrects and parcels volume images. Finally, the segmentation labels were defined at every voxel on the T1-weighted image of each subject. To achieve correspondence between T1 and PET images of each subject, the PET image was then co-registered to the T1-weighted image transformed by FreeSurfer using FSL linear registration (FMIRB Software Library, FLIRT). In this step, 11 subjects were excluded due to errors in preprocessing (Freesurfer failed to produce the result in one subject, and ten subjects had errors in co-registration of PET scans and T1 MR images).

In line with the RCTU scoring method in VA, we modelled our scoring protocol based on axial images. Since the raters inspected five regions (lateral temporal lobe, frontal lobe, posterior cingulate/precuneus, parietal cortex, and striatum) in both hemispheres, we tried to select the corresponding regions of interest on axial slices.

To select slices containing the regions of interest, we first defined the top and the bottom slices using the relative locations, especially height, with tissues in the segmented image. Specifically, a slice corresponding to the top of the lateral ventricle was selected as the top slice (The slice number was referred to as *SN*_top_). To obtain the slice number of the bottom slice (*SN*_bottom_), we selected a slice closest to the centre of mass of the temporal lobe (inferior-temporal, middle-temporal, superior-temporal, temporal-pole, transverse temporal, bankssts, fusiform, entorhinal, parahippocampal). After defining these margins, the top slice was defined as the first slice (slice number *S*_*1*_ *= SN*_top_), and this slice was suitable for assessing the parietal cortex. The slice number of the second slice (*S*_*2*_) was calculated as *S*_*2*_ = *SN*_top_ + 0.25 × (*SN*_bottom_ − *SN*_top_), and this slice was appropriate for assessing the posterior cingulate/precuneus. To select the third slice (slice number S_3_) for evaluation of the frontal cortex, the slice number was estimated as *S*_*3*_ = *SN*_top_ + 0.5 × (*SN*_bottom_ − *SN*_top_). Then the bottom slice was defined as the fourth slice (slice number *S*_*4*_ *= SN*_bottom_) and was suitable for analysing the lateral temporal lobe. For the striatum, we selected the slice (slice number *S*_*5*_) closest to the centre of mass of regions containing the caudate nucleus and putamen **(**Fig. 1a**)**.

### Hierarchical amyloid staging

#### Step 1. Cortical amyloid positivity classification

From the PET slices for a subject, we extracted the target ROIs using parcellation with FreeSurfer analogously to the areas mainly examined by human raters. For each slice, we obtained ROIs from both right and left sides, resulting in 10 ROIs (eight cortical and two striatal regions) per subject.

For characterisation of amyloid tracer uptake, we defined an uptake curve feature for each cortical region. This curve shows the intensity of uptake according to the location from the grey matter to the white matter (Fig. 1b). To calculate this curve feature, we applied the fast-marching [15] algorithm to the extracted regions using grey matter as a seed for distance. That is, a smaller distance value indicates greater proximity to the grey matter. We discretised the distance from the grey matter into 15 bins. We used the values of the first 12 bins for our intensity curve feature since it was enough for RCTU prediction. Instead of using the raw PET intensity, we normalised slices by dividing the intensity values by the mean intensity of all pixels within eight cortical ROIs.

Elements in our proposed feature have multicollinearity, and the target labels, the RCTU scores, have ordinal property. Considering these properties of the variables, we used the support vector machine (SVM) regression model for RCTU prediction (Fig. 1c). For each region (*k* = 1 … 8) per subject (*i = 1 … n*), we set:

$$ {y}_i^k=\mathrm{RCTU}\ \mathrm{variable}\ \left\{1,2,3\right\} $$ (Dependent variable).

$$ {x}_i^k=\mathrm{the}\ \mathrm{intensity}\ \mathrm{curve}\ \mathrm{feature}\ \mathrm{vector} $$ (Independent variable).

With these variables, the regressor was constructed using the training set (for all subjects except a test subject), and the pRCTU for the test subject was calculated from the regressor. This process was performed for all subjects and all cortical ROIs.

For amyloid positivity classification (Fig. 1d), we used the SVM algorithm, which is a supervised classification algorithm that has been well-validated and applied to binary classification approaches in neuroimaging studies. In the present study, the hyperplane seeks to divide the feature space (such as the pRCTU based on eight regions concentrated by a clinician) for each class or label (such as amyloid-negative or amyloid-positive status) optimally. We used a built-in Matlab function fitrsvm(…) and fitcsvm(…) for SVM regression and classification, respectively, with default parameters (Linear kernel, Kenel scale parameter: 1, Box Constrain: 1, Kernel offset parameter: 0, Half the width of epsilon-insensitive band: 13.49, SMO solver) from Statistics and Machine Learning Toolbox (https://www.mathworks.com/help/stats/index.html).

To estimate the performance of step 1, we applied the leave-one-out cross-validation scheme to our problem. This scheme uses one subject as a test set, with the other subjects in a training set. By changing this pair, we evaluated every subject and measured performance scores such as accuracy, sensitivity, specificity, and area under the curve (AUC). In training phases, the cortical curve features of all cortical ROIs were extracted from the training subjects. Then, we modelled the regressor from the training feature set and extracted pRCTU from the regressor. Lastly, the classifier constructed a relationship between the vector of pRCTU values and the positivity label from the training dataset. For testing, we used the cortical curve feature from the test subject.

#### Step 2. Striatal positivity classification

To characterise striatal tracer uptake, we first aligned the *S*_*5*_ slice to the MNI 152 template slice by non-linear registration to ensure that the striatum of every subject was located in the same pixel position. Then, we defined the uptake values of all pixels within the striatal region as features (Fig. 1e).

**Fig. 2:**
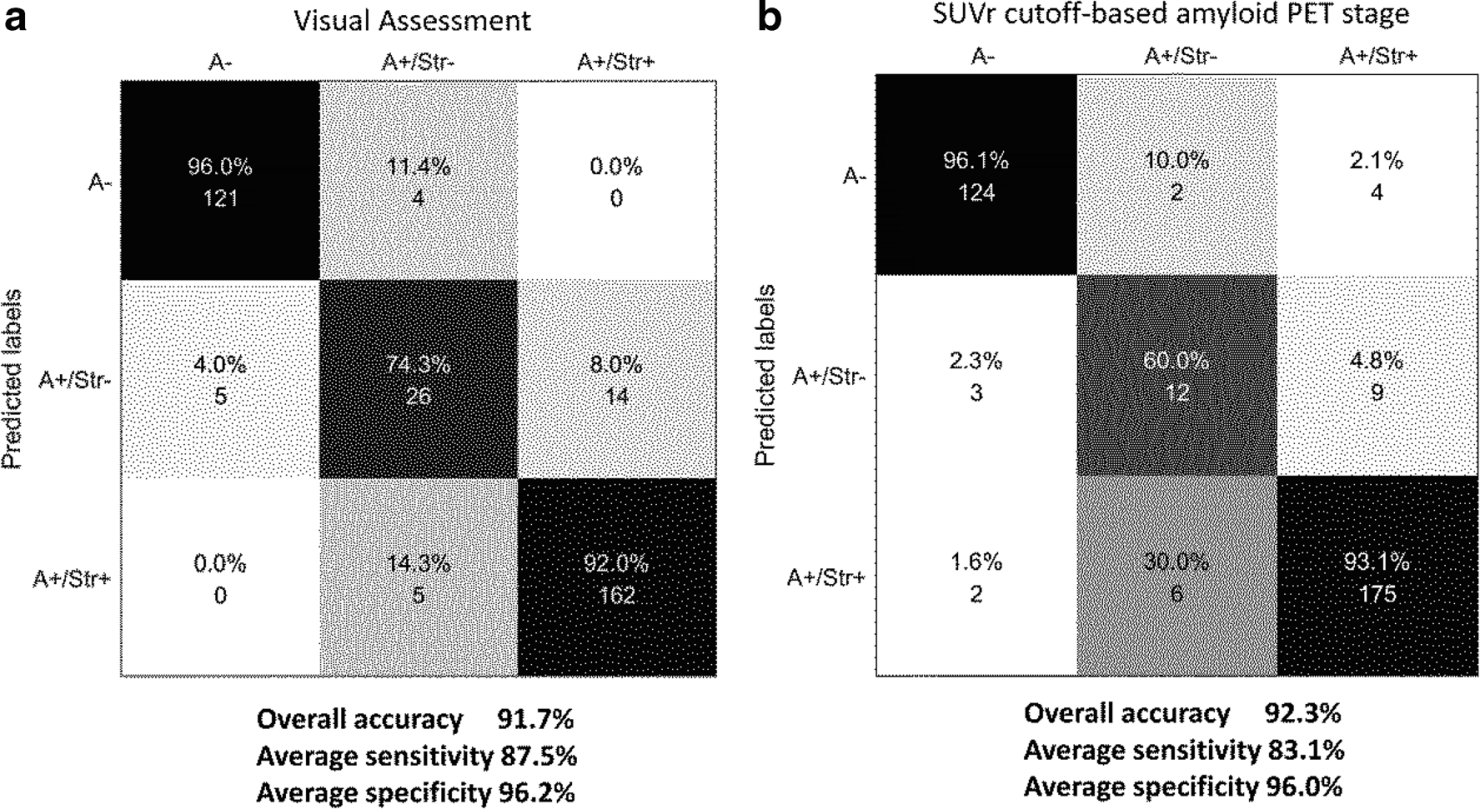
Confusion matrix showing the final classification results. **a** The visual assessment and **b** SUVr cutoff-based amyloid stage were used as standard of truth. For overall classification accuracy, the number of correctly classified subjects was divided by the total number of subjects. Average sensitivity (specificity) was derived by averaging sensitivities (specificities) for the three stages. SUVr standardised uptake value ratio, A− amyloid PET-negative, A+ amyloid PET-positive, Str− negative striatal uptake, Str+ positive striatal uptake

For individuals classified as amyloid-positive in step 1, we applied principal component analysis (PCA) and a linear discriminant analysis (LDA) classification approach to the uptake intensity of pixels in the striatum for further staging (Fig. 1f). The feature vector characterising the striatal region was high-dimensional, possessing 1469 elements. We used the PCA and LDA for dimensional reduction and inter-group separation, respectively. Similarly to step 1, we used leave-one-out cross-validation scheme and evaluated the classification performance. Through these two classification steps, we could determine each subject’s amyloid PET stage as stage 0 (amyloid-negative), stage 1 (cortical amyloid-positive and striatal amyloid-negative), or stage 2 (cortical- and striatal amyloid-positive) (Fig. 2).

### Discriminative pattern analysis

In order to analyse relative importance of imaging features in each classifier, we extracted the discriminative patterns using the weight vector of the classifiers [16]. For step 1, the discriminative pattern represents the relative contributions of eight brain regions in discriminating cortical positivity, while for step 2, it represents the topographic pattern of contributions in discriminating striatal positivity. Specifically for step 1, the relative contribution (*D*_*a*+_) of imaging features was computed using the coefficients of the orthogonal vector to the hyperplane of SVM [17]. The discriminative pattern (*D*_*s*+_) of step 2 was constructed using the weight vector of the PCA and LDA models (*w* = *M*_*PCA*_ × *M*_*LDA*_) [18]. Each discriminative pattern was normalised by its maximum absolute value to 0~1 for synchronisation of the colourisation scale.

### Neuropsychological tests

For a comprehensive assessment of cognitive function, the Korean version of the mini-mental state examination (K-MMSE) and the Seoul Neuropsychological Screening Battery, 2nd edition (SNSB-II) were used. [19] The SNSB-II measures multiple cognitive functions, including attention (forward and backward digit span), language (repetition, calculation, ideomotor apraxia test, and the Korean version of the Boston Naming Test), visuospatial function (Rey Complex Figure Test: copying), memory (Seoul Verbal Learning Test: immediate recall, delayed recall, and recognition; Rey Complex Figure Test: immediate recall, delayed recall, and recognition), and executive function (contrasting program, go-no-go test, phonemic and semantic Controlled Oral Word Association Test, and the Korean version of the Stroop Color and Word Test) [20]. For comparisons between groups, the composite scores for five cognitive domains (attention, memory, language, visuospatial function, and frontal/executive function) were used. The composite score for attention was defined as the sum of the forward and backward digit span scores. For language, visuospatial, memory, and frontal/executive domains, the composite score was defined as the average of the standard scores of the tests corresponding to each domain.

### Measurement of cortical thickness and hippocampal volume

For comparison of cortical thickness and hippocampal volume (HV) between stages, T1-weighted MR images were automatically processed using the standard Montreal Neurological Institute image processing software (CIVET). This software has been well-validated and is extensively described elsewhere, including in aging/atrophied brain studies [21, 22]. To measure HV at baseline, we used an automated hippocampus segmentation method using the graph-cut algorithm combined with atlas-based segmentation and morphological opening as described in an earlier study [23].

### Statistics

Continuous variables were expressed as mean (standard deviation (SD)), and categorical variables were expressed as *N* (%). In terms of classification performance, we first calculated overall classification accuracy, average sensitivity, and specificity in predicting VA labels using our model. In addition, we trained another model using the same pipeline only by changing the standard of truth from VA to SUVr cutoff-based amyloid stage and evaluated its prediction performance. We compared the neuropsychological parameters among groups by using analysis of covariance (ANCOVA) tests after controlling for age, sex, and years of education. For MRI parameters (cortical thickness and HV), we additionally controlled for intracranial volume (ICV). In addition to pairwise comparisons, we tested for linear trends with linear contrast analysis. Multiple linear regression analysis was performed to assess the associations between mean pRCTU and neuropsychological/structural parameters, after controlling for age, sex, and ICV for MRI variables and age, sex, and years of education for neuropsychological variables. For outcome variables showing significant association with both stage and mean pRCTU value, path analysis was performed to examine whether there is any mediation effect between predictors. For path analysis, we used the *lavaan* (version 0.6–3) package in R. All statistical analyses were performed using R version 3.5.3.

## Results

### Clinical characteristics

Scans of 337 subjects were finally included in the analysis. The characteristics of the participants are shown in Table 1. The mean (SD) age of all participants was 70.5 (9.2) years (range, 33 to 88 years). Fifty (14.8%) subjects were CN, 145 (43.0%) subjects were diagnosed with amnestic MCI, and 142 (42.1%) subjects had dementia. On visual assessment, 126 (37.3%) subjects were negative for amyloid deposition (Stage 0 by VA). Among the 211 subjects who showed positive cortical florbetaben uptake, 35 (10.4%) did not show significant uptake in the striatum (Stage 1 by VA) while 176 (52.2%) showed both cortical and striatal uptake (Stage 2 by VA).

**Table 1:** Clinical and imaging characteristics of participants (*N* = 337)

Age, years	70.5 (9.2)
Female sex, No. (%)	165 (49.0)
Education, years	12.5 (4.6)
MMSE	23.1 (6.1)
Diagnosis	
CN, No. (%)	50 (14.8)
MCI, No. (%)	145 (43.0)
AD dementia, No. (%)	142 (42.1)
VA of amyloid PET	
No cortical uptake	126 (37.4%)
Cortical uptake only	35 (10.4%)
Cortical and striatal uptake	176 (52.2%)

*MMSE* mini-mental status examination, *CN* cognitively normal, *MCI* mild cognitive impairment, *AD* Alzheimer’s disease, *VA* visual assessment, *PET* positron emission tomography

### Performance in florbetaben PET staging and quantification

In step 1, the pRCTU values of eight cortical ROIs were used for both quantification and classification. First, these pRCTU values were averaged to represent a quantification value of cortical tracer uptake, which ranged from 0.70 to 3.55 and correlated well with global SUVr (Pearson’s *R* = 0.9, *p* < 0.001). Next, the eight RCTU values were combined into a feature vector and entered into the SVM classifier to determine amyloid positivity. As a result, the subjects were classified into three stages, and the number of subjects in each stage was 125, 45, and 167 for stage 0, 1, and 2, respectively. The accuracy, sensitivity, and specificity in discriminating amyloid-positive from amyloid-negative subjects (Stage 0 vs. Others) were 97.3%, 98.6%, and 95.2%, respectively (AUC = 0.992) (Online Resource 1A).

In step 2, among subjects with a positive cortical amyloid deposit, the striatal amyloid positivity was determined with 91.1% accuracy, 90.0% sensitivity, and 92.1% specificity (AUC = 0.963, Online Resources 1A). When we applied the SVM classification for the same task, the classification performance was similar in terms of AUC (LDA 0.963, SVM 0.947) and accuracy (LDA 91.1%, SVM 91.6%). Figure 2 a shows a confusion matrix of the overall classification. The overall classification accuracy, indicating the probability of a single subject being classified correctly into one of the three stages, was 91.7% with 96.2% specificity and 87.5% sensitivity.

When the prediction model was trained using the SUVr cutoff-based labels as a standard of truth, the overall accuracy of prediction was 92.3%, with 83.1% sensitivity and 96% specificity (Fig. 2b, Online resources 1B). Of note, the proportion of overall agreement between the two standards of truth was 91.4% (96.1% for step 1 and 91.2% for step 2).

### Visualisation of discriminative pattern

**Fig. 3:**
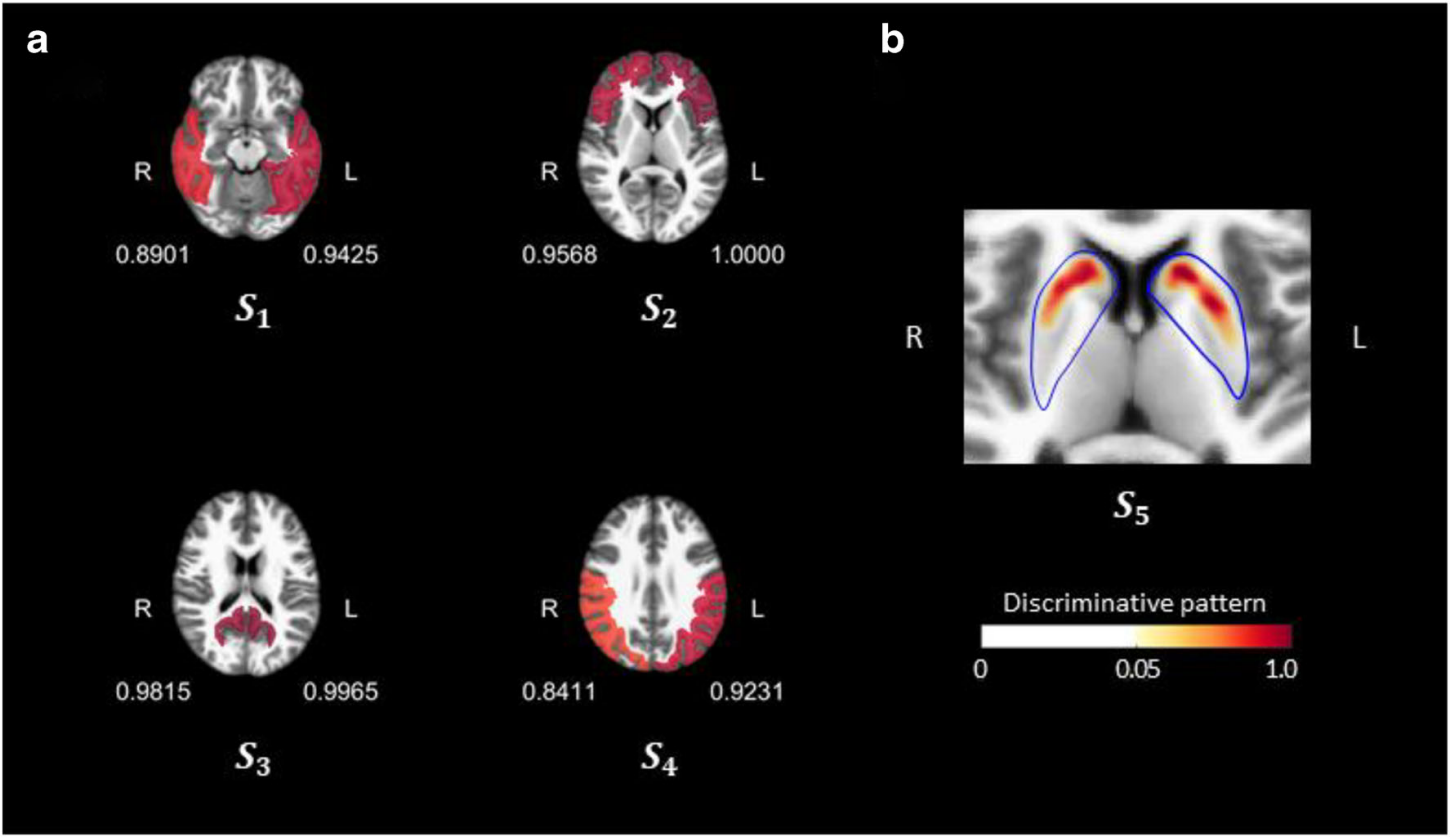
The discriminative scores (**a**) and pattern (**b**) visualised on axial slices. For each classifier, scores representing the relative contribution in discriminating positive from negative cases were normalised by the maximum value, resulting in a 0 to 1 scale

For visualisation, we mapped *D*_*a*+_(from step 1) on the template slice images (*S*_1_, *S*_2_, *S*_3_, *S*_4_), with a yellow-red colour scale representing the importance of the feature for the classification of cortical uptake positivity (more red colour indicates a greater contribution (Fig. 3a)). For the classification of striatal positivity, the *D*_*s*+_ (from step 2) was coloured on the striatum slice (*S*_5_), also using the same colour scale where red indicated a greater contribution (Fig. 3b).

Step 1 included eight ROIs, and each ROI had a single discriminative score. All eight regions had positive scores, meaning that all regions contributed to classifying scans as positive in step 1 (Fig. 3a). Within the 8 ROIs, the left frontal and left precuneus regions showed the highest discriminative scores, followed by the right precuneus and right frontal regions. The right parietal region showed the lowest discriminative score.

In step 2, since we used the uptake intensity of all pixels within the striatal ROI, we could obtain the regional discriminative pattern (Fig. 3b). The caudate nucleus and anterolateral portion of the putamen contributed to classifying scans into stage 2.

### Post hoc assessment of misclassified subjects

**Fig. 4:**
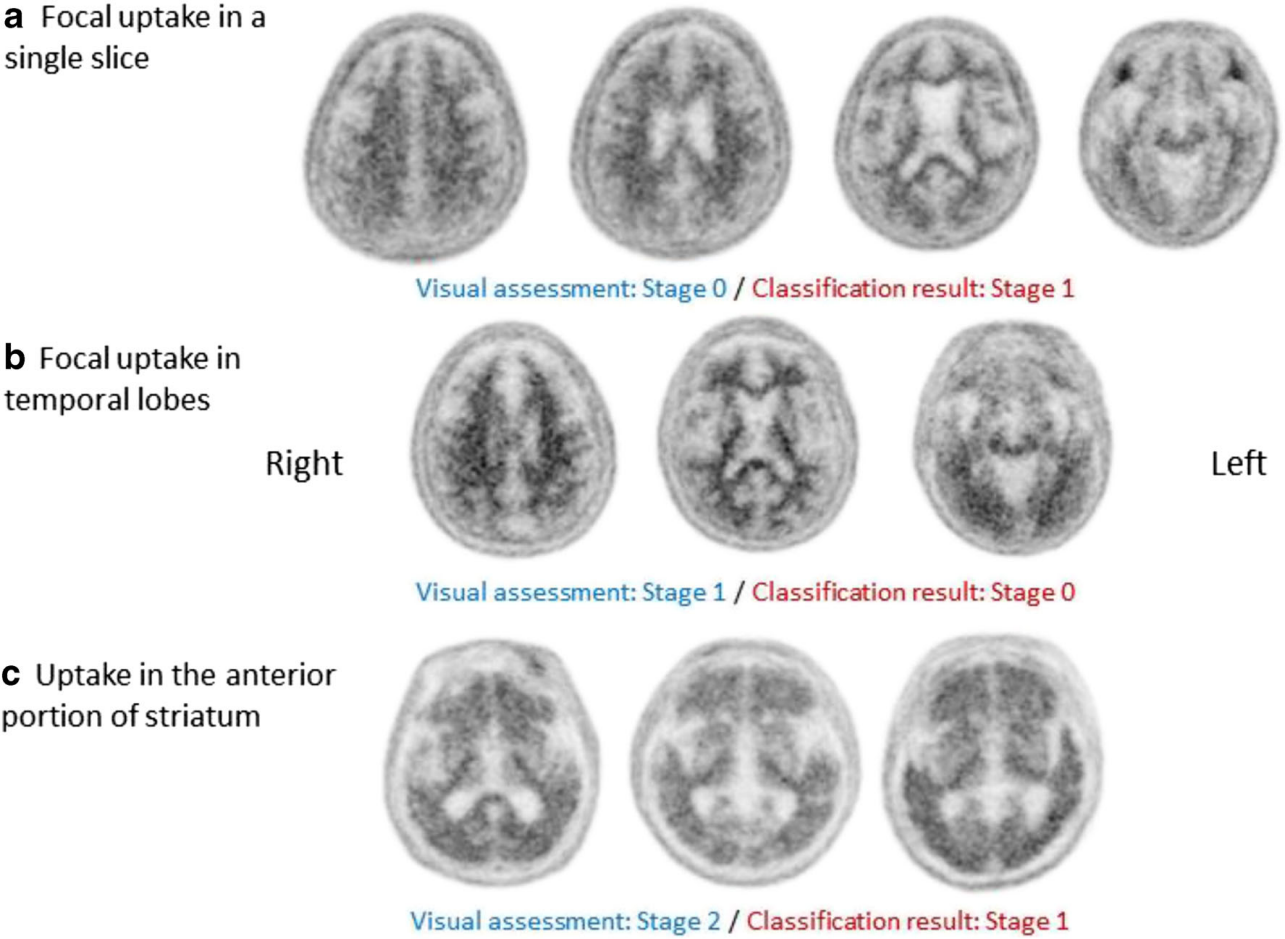
Representative images of misclassified subjects. (a) A subject with marginal uptake in the right parietal lobe (the leftmost figure), which is not evident in the next slice (second figure from the left). (b) A subject who had focal uptake in both (more prominent in the left) temporal lobes. (c) Three subjects with higher florbetaben uptake in the anterior portion of the striatum compared with the posterior portion

We performed a post hoc assessment for misclassified subjects. A total of 28 classifications did not match the VA. Five subjects who were visually rated as stage 0 were classified as stage 1 by our classifier (Fig. 4a). Although the reason for these misclassifications was not clear, the fact that our classifier analysed only one slice for each ROI, while human raters observed multiple slices, might have had some impact. Four misclassified subjects who were visually rated as stage 1 but classified as stage 0 by our classifier tended to have focal florbetaben uptake, especially in the temporal region (Fig. 4b). Fourteen scans, the majority of the misclassified scans, were visually read as stage 2, while our classifier classified them as stage 1. These subjects tended to have higher florbetaben uptake in the anterior than in the posterior portion of the striatum (Fig. 4c). Only five cases showed the opposite pattern (stage 1 by VA, stage 2 by the classifier).

### Structural and clinical implications of the determined stages and mean pRCTU

#### Impact on structural MRI parameters

**Fig. 5:**
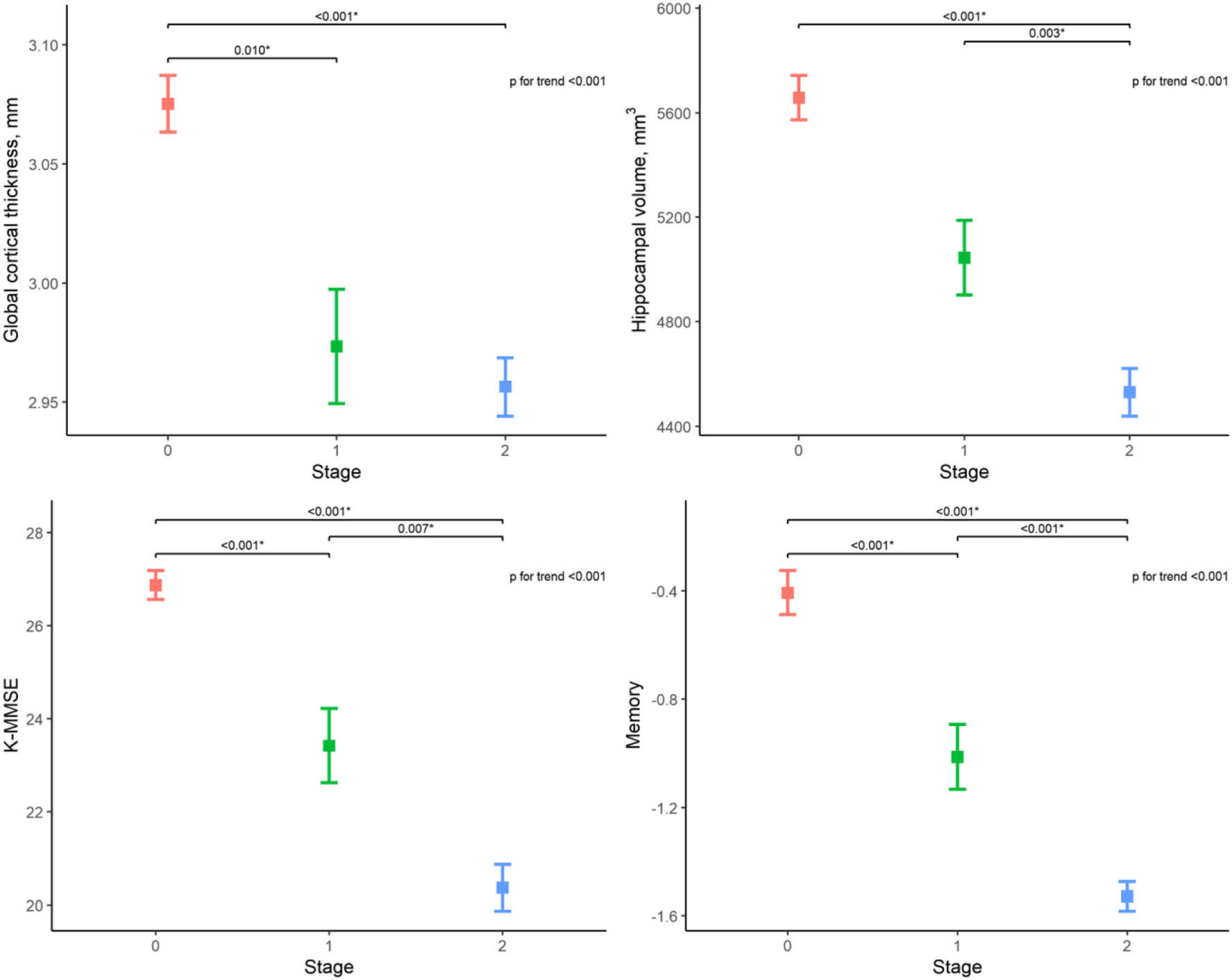
Comparisons of structural MRI parameters and clinical scores between the determined stages. Error bars indicate standard errors. The *p* values from comparisons between stages are indicated at the top of each plot. Bonferroni correction was performed for multiple group comparison. K-MMSE Korean version of mini-mental state examination

All MRI parameters of stage 2 subjects were worse than those of stage 0 subjects (*p* < 0.001 for all variables), and the scores showed a clear decreasing trend with advancing stage (*p* for trend < 0.001 for all variables). Specifically, cortical thickness in the parietal (*p* = 0.006), temporal (*p* = 0.009), and occipital (*p* = 0.044) lobes and global cortical thickness (*p* = 0.010) were significantly lower in stage 1 compared with those in stage 0. Within the amyloid-positive group, subjects in stage 2 showed a lower HV (*p* = 0.003) compared to those in stage 1 (Fig. 5). Online Resource 2 shows the detailed results for all structural and neuropsychological variables. The mean pRCTU showed significant negative associations with all structural parameters when all subjects were included (*p* < 0.001 for all variables)(Table 2A). The negative association persisted for the parietal (*p* = 0.014), temporal (*p* = 0.002), and global (*p* = 0.023) cortical thickness within amyloid-positive (stages 1 and 2) subjects.

#### Impact on neuropsychological performance scores

Neuropsychological test scores of amyloid PET stage 2 subjects were lower than those of stage 0 subjects (*p* < 0.001 for all variables). The K-MMSE score (*p* < 0.001) and the composite memory (*p* < 0.001), visuospatial (*p* = 0.023), and frontal executive function scores (*p* < 0.001) were significantly lower in stage 1 compared to those in stage 0. Within the amyloid-positive group, subjects in stage 2 showed lower K-MMSE (*p* = 0.007) and composite memory scores (*p* < 0.001) than those in stage 1. Like structural parameters, all neuropsychological variables showed a decreasing trend with advancing stage (*p* < 0.001). Multiple linear regression analyses showed a significant negative relationship between mean pRCTU and all neuropsychological variables (*p* < 0.001 for all variables) regardless of the inclusion of amyloid-negative (stage 0) subjects (Table 2B).

#### Evaluation of the direct vs. indirect effect of amyloid PET stage on outcome variables

**Fig. 6:**
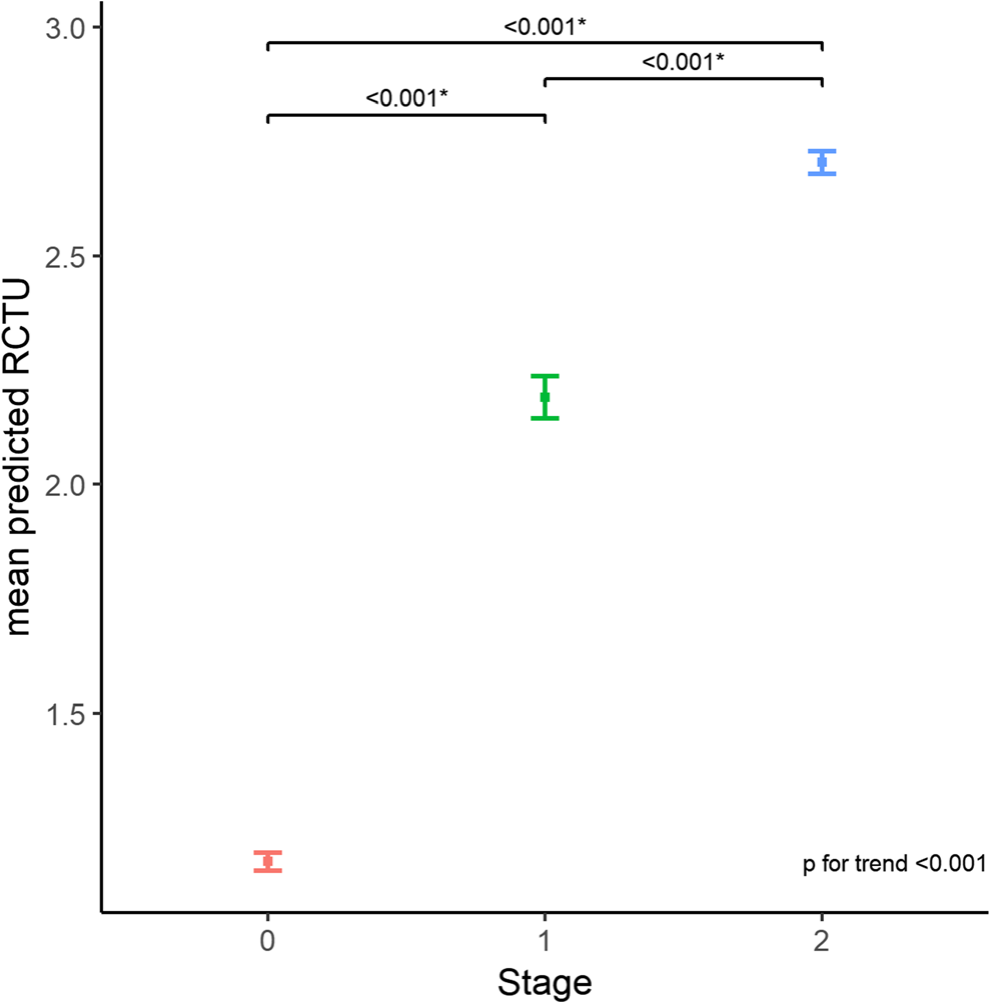
Mean pRCTU values according to the amyloid PET stage. Error bars indicate standard errors. pRCTU predicted regional cortical tracer uptake

**Fig. 7:**
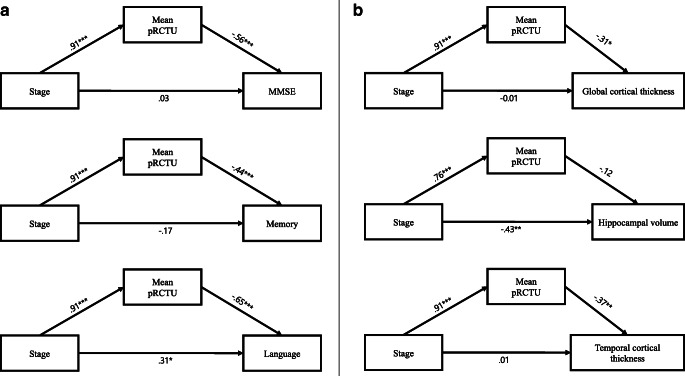
Diagrams of path analysis. Standardised coefficients are presented on the path. The models for neuropsychological variables were controlled for age, sex, and education, and the models for structural MRI variables were controlled for age, sex, and intracranial volume. *p < 0.05; **p < 0.01; ***p < 0.001. pRCTU predicted regional cortical tracer uptake

As shown in Fig. 6, there were significant differences in the mean pRCTU value between groups (*p* < 0.001 for all combinations), and the mean pRCTU showed a clear increasing tendency with advancing stage (*p* for trend < 0.001). The results from path analysis suggested complete mediation of mean pRCTU in relationships between the stage and all neuropsychological outcomes (Fig. 7a). Although the direct effect of stage on the language function (standardised coefficient = 0.31, *p* = 0.17) is significant, the negative total effect is driven by mean pRCTU, as the direct effect was positive in direction. Complete mediation was also observed in relationships between stage and MRI variables, except for the frontal cortical thickness (partial mediation) and HV (no mediation) (Fig. 7b).

**Table 2:** Results from linear regression analysis between mean pRCTU and neuropsychological/structural parameters

A. Structural parameters																		
	Frontal cortex			Parietal cortex			Temporal cortex			Occipital cortex			Global cortex			Hippocampal volume		
	*B* (SE)	*p*	Adj. *R*^2^	*B* (SE)	*p*	Adj. *R*^2^	*B* (SE)	*p*	Adj. *R*^2^	*B* (SE)	*p*	Adj. *R*^2^	*B* (SE)	*p*	Adj. *R*^2^	*B* (SE)	*p*	Adj. *R*^2^
All subjects	− 0.042 (0.010)	< 0.001*	0.196	− 0.082 (0.012)	< 0.001*	0.238	− 0.092 (0.012)	< 0.001*	0.279	− 0.068 (0.012)	< 0.001*	0.215	− 0.066 (0.010)	< 0.001*	0.261	− 598.9 (75.7)	< 0.001*	0.298
Subjects in stage 1 and stage 2	− 0.035 (0.029)	0.232	0.127	− 0.087 (0.035)	0.014*	0.142	− 0.115 (0.036)	0.002*	0.137	− 0.062 (0.033)	0.06	0.089	− 0.067 (0.029)	0.023*	0.134	− 264.5 (214.5)	0.219	0.104
B. Neuropsychological parameters																		
	K-MMSE			Attention			Language			Visuospatial			Memory			Frontal/executive		
	*B* (SE)	*p*	Adj. *R*^2^	*B* (SE)	*p*	Adj. *R*^2^	*B* (SE)	*p*	Adj. *R*^2^	*B* (SE)	*p*	Adj. *R*^2^	*B* (SE)	*p*	Adj. *R*^2^	*B* (SE)	*p*	Adj. *R*^2^
All subjects	− 4.266 (0.363)	< 0.001*	0.345	− 0.858 (0.171)	< 0.001*	0.223	− 0.382 (0.056)	< 0.001*	0.142	− 0.718 (0.085)	< 0.001*	0.252	− 0.739 (0.056)	< 0.001*	0.362	− 0.721 (0.071)	< 0.001*	0.313
Subjects in stage 1 and stage 2	− 4.994 (1.081)	< 0.001*	0.205	− 1.692 (0.436)	< 0.001*	0.246	− 0.707 (0.169)	< 0.001*	0.08	− 1.143 (0.255)	< 0.001*	0.197	− 0.656 (0.134)	< 0.001*	0.164	− 0.900 (0.194)	< 0.001*	0.212

Models with neuropsychological outcomes included age, sex, and years of education as covariates. Models with structural outcome variables included age, sex, and total intracranial volume as covariates

*pRCTU* predicted regional cortical tracer uptake, *K-MMSE* Korean version of mini-mental status examination, *Adj. R*^*2*^ adjusted *R* squared

The same path analysis was also performed within amyloid-positive (stage 1 and stage 2) subjects by using K-MMSE and composite memory scores as outcomes that showed significant association with both pRCTU and amyloid PET stage. The results also showed the complete mediation effect of mean pRCTU values (Online Resource 3).

## Discussion

We developed a machine learning–based classifier for in vivo amyloid staging by assessment of cortical and striatal tracer uptake. This classifier showed excellent accuracy in discriminating between amyloid-positive and amyloid-negative subjects (97.3%) and between subjects with and without striatal involvement (91.1%). The in vivo amyloid stage and the mean pRCTU both correlated well with clinical and structural outcome variables. Especially, the clinical and structural impact of the PET stage was largely mediated by the mean pRCTU. Thus, our findings outline the clinical impact of PET parameters obtained with the machine learning algorithm, presenting a new perspective for utilisation of amyloid PET imaging.

From the standpoint of methodological development, we were able to classify the in vivo amyloid stage using our machine learning-based algorithm. We proposed new methods for slice selection and feature definition, which showed excellent performance. In contrast to the SUVr cutoff method, reference contamination in the cerebellum does not influence our results since our feature vector characterises the pattern of uptake from the cortex to subcortical white matter. Consistent with previous machine learning studies [3, 4], our classifier (step 1) showed similarly high classification performance in discriminating positive and negative amyloid scans. However, while those studies focused on positive/negative classification, we further classified amyloid-positive subjects into two distinct stages according to the striatal involvement. Recent studies on amyloid PET staging have attempted staging using threshold values proposed in each study [7–9]. In line with those study results, our results also showed that subjects with positive striatal uptake had a worse clinical and structural profile. In particular, HV was significantly lower in stage 2 than in stage 1, which might have led to the lower memory scores in stage 2.

In the present study, we also developed an automatic quantification method for cortical tracer uptake using the mean pRCTU value, which is obtained in the process of positive/negative classification. The mean pRCTU, which represents the cortical amyloid burden, correlated well with global SUVr and was also well-associated with neuropsychological and MRI parameters. The association between PET parameters and outcome variables was evident in the whole group. More notably, within the amyloid-positive subjects, the mean pRCTU showed correlation with non-memory cognitive functions and cortical thickness, while no differences in these variables were observed between stage 1 and stage 2. Although the amyloid PET images have been interpreted based on the all-or-none concept, our findings suggest that additional information such as stage or quantification measures that our algorithm provides can also be informative.

Another noteworthy finding was that the detrimental effect of a higher stage on clinical outcomes was largely mediated by an increased cortical amyloid burden. Although results from recent studies suggest that striatal involvement of amyloid deposition is associated with worse outcomes [7, 8], it is not clear whether this association was driven by the striatal amyloid itself. The striatum is a part of the frontal-striatal circuit and is significantly associated primarily with frontal executive and behavioural functions [24]. However, patients with striatal involvement showed lower performance in memory assessments or lower HV than those without striatal involvements. It is noticeable that, consistent with a previous study [9], our study showed a significant correlation between advanced stage and increased cortical amyloid. More recent studies also showed that the amyloid PET stages based on cortical tracer uptake were associated with cognitive impairments [25, 26]. Therefore, advanced stage may lead to increased cortical uptake, resulting in worse clinical outcomes. In one of the studies on in vivo amyloid staging, the amyloid PET stage, compared with the cortical amyloid burden, showed a greater impact on the decline in the MMSE score and the baseline HV [8]. In contrast, our path analysis results showed that striatal uptake was only indirectly associated with most clinical/structural outcomes, and these relationships were completely mediated by the quantification marker of cortical uptake. Regarding HV, however, striatal involvement was directly associated without a significant mediation effect of pRCTU. We can cautiously suggest that cortical amyloid deposition plays a more important role than striatal amyloid deposition in deterioration of cognitive function and progression of brain atrophy. This issue needs to be further investigated and discussed in future studies.

Discriminative scores in step 1 showed that while all ROIs significantly contributed to the classification, the frontal and posterior cingulate/precuneus areas had the highest discriminative scores, followed by the lateral temporal and parietal regions. This finding is in line with a previous report in which early Aβ accumulation started in the precuneus, posterior cingulate, and medial orbitofrontal cortices [27]. In another previous study on machine learning classification, the precuneus, striatum, posterior and midcingulate and anteromedial frontal cortex showed high contributions to the classification [3]. For striatal amyloid positivity, we could obtain a more region-specific discriminative pattern since we used the feature vector representing uptake values of all pixels within the striatal ROI. In step 2, the anterolateral region of the putamen contributed significantly in determining stage 2. This result is consistent with a previous autopsy study in which amyloid pathology was more prominent in the ventral than the dorsal striatum [28].

The strength of our study was that we developed a machine learning algorithm for amyloid staging with relatively larger sample size compared with previous studies. However, this study has some limitations. First, since the purpose of this study was to replicate the VA of tracer uptake, the classification results might not predict the actual amyloid pathology. Second, since this is a cross-sectional study, we could not evaluate the impact of PET parameters on the longitudinal outcome. Third, since we used only four slices for positive/negative classification, some focal uptake may have been missed in the unselected slices or regions. Fourth, our method uses both PET and MR images for automatic RCTU prediction, which, therefore, could be partially limited in clinical applications. The MR image, however, is used only for accurately extracting the location of the lateral ventricle. Thus, the proposed method could be easily extended to exploit only PET images without using MR images for reference. Finally, another obstacle to direct adoption of our technique into clinical practice is the need for identical MRI and PET protocols for the training set and any new patient to be classified. Nevertheless, it is noteworthy that our new classification and quantification method for amyloid PET images showed excellent accuracy and significant correlation with clinical outcomes, providing some new perspectives on striatal and cortical amyloid. Our future studies will focus on developing computationally more efficient and generalisable methods with comparable or better classification accuracy.

In conclusion, using a machine learning algorithm, we achieved high accuracy for in vivo amyloid PET staging. The in vivo amyloid stage was associated with cognitive function and cerebral atrophy mostly through the mediation effect of cortical amyloid. In clinical practice, this proposed classifier might facilitate biomarker-supported diagnosis as well as in vivo staging and quantification of amyloid pathology of AD patients.

## Electronic supplementary material

ESM 1 (DOCX 1498 kb)

ESM 2 (DOCX 30 kb)

ESM 3 (DOCX 97 kb)
