# Supplementary material for: Staging and quantification of florbetaben PET images using machine learning: impact of predicted regional cortical tracer uptake and amyloid stage on clinical outcomes
Source: Eur J Nucl Med Mol Imaging. 2019 Dec 28;47(8):1971–83. doi: 10.1007/s00259-019-04663-3 (PMC7299909; doi:10.1007/s00259-019-04663-3)
Supplement: Supplementary file 2 — (DOCX 30 kb) [file 259_2019_4663_MOESM2_ESM.docx]

**Staging and quantification of florbetaben PET images using machine learning: Impact of predicted regional cortical tracer uptake and amyloid stage on clinical outcomes**

Jun Pyo Kim^1,2,3*^, Jeonghun Kim^4*^, Yeshin Kim^9^, Seung Hwan Moon^10^, Yu Hyun Park^1,2^, Sole Yoo^11^, Hyemin Jang^1,2,3^, Hee Jin Kim^1,2,3^, Duk L. Na^1,2,3,6^, Sang Won Seo^1,2,3,7,8†^, Joon-Kyung Seong^4,5†^

* These authors contributed equally to this work

†These authors contributed equally to this work

^1^Department of Neurology, Samsung Medical Center, Seoul, Korea

^2^Samsung Alzheimer Research Center, Samsung Medical Center, Seoul, Korea

^3^Neuroscience Center, Samsung Medical Center, Seoul, Korea

^4^Department of Bio-convergence Engineering, Korea University, Seoul, Korea

^5^School of Biomedical Engineering, Korea University, Seoul, Korea

^6^Department of Health Sciences and Technology, SAIHST, Sungkyunkwan University, Seoul, Korea

^7^Department of Clinical Research Design & Evaluation, SAIHST, Sungkyunkwan University, Seoul, Korea

^8^Center for Clinical Epidemiology, Samsung Medical Center, Seoul, Korea

^9^Kangwon National University Hospital, Chuncheon, Korea

^10^Department of Nuclear Medicine, Samsung Medical Center, Seoul, Korea

^11^Department of Cognitive Science, Yonsei University, Seoul, Republic of Korea

**Address for correspondence:**

Joon-Kyung Seong, PhD

School of Biomedical Engineering, Korea University

145 Anam-ro, Seongbuk-gu, Seoul, Republic of Korea

Tel: +82-2-3290-5660, Fax: +82-2-921-6434

Email: jkseong@korea.ac.kr

Sang Won Seo, MD PhD

Department of Neurology, Samsung Medical Center, Sungkyunkwan University, School of Medicine,

81 Irwon-ro, Gangnam-gu, Seoul, 06351, South Korea

Tel.: 82-2-3410-1397, Fax: 82-2-3410-0052

Email: [sangwonseo@empal.com](mailto:sangwonseo@empal.com)

[sangwonseo@empal.com](mailto:sangwonseo@empal.com)

Online Resource 2. Group comparison of structural MRI parameters and clinical scores

|  | Stage 0 | Stage 1 | Stage 2 | *p* (0 vs. 1) | *p* (0 vs. 2) | *p* (1 vs. 2) | *p for trend* |
| --- | --- | --- | --- | --- | --- | --- | --- |
| Structural |  |  |  |  |  |  |  |
| Cortical Thickness | |  |  |  |  |  |  |
| Frontal | 3.11(0.13) | 3.02(0.17) | 3.03(0.15) | 0.063 | <0.001^*^ | 1.000 | <0.001^*^ |
| Parietal | 3.04(0.13) | 2.94(0.18) | 2.90(0.20) | 0.006^*^ | <0.001^*^ | 1.000 | <0.001^*^ |
| Temporal | 3.27(0.14) | 3.15(0.19) | 3.11(0.20) | 0.009^*^ | <0.001^*^ | 0.445 | <0.001^*^ |
| Occipital | 2.91(0.18) | 2.80(0.16) | 2.79(0.18) | 0.044^*^ | <0.001^*^ | 1.000 | <0.001^*^ |
| Global | 3.08(0.13) | 2.97(0.16) | 2.96(0.16) | 0.010^*^ | <0.001^*^ | 1.000 | <0.001^*^ |
| HV | 7438.6(1275.4) | 6671.0(1441.1) | 6040.5(1635.7) | 0.069 | <0.001^*^ | 0.003^*^ | <0.001^*^ |
|  |  |  |  |  |  |  |  |
| Clinical |  |  |  |  |  |  |  |
| K-MMSE | 26.9(3.4) | 23.4(5.4) | 20.4(6.5) | <0.001^*^ | <0.001^*^ | 0.007^*^ | <0.001^*^ |
| Attention | 9.8(2.6) | 9.2(2.0) | 8.8(2.7) | 0.895 | <0.001^*^ | 0.327 | <0.001^*^ |
| Language | -0.04(0.53) | -0.41(0.81) | -0.52(0.93) | 0.108 | <0.001^*^ | 0.457 | <0.001^*^ |
| Visuospatial | 0.01(0.76) | -0.49(1.26) | -1.02(1.50) | 0.023^*^ | <0.001^*^ | 0.135 | <0.001^*^ |
| Memory | -0.41(0.90) | -1.01(0.80) | -1.53(0.70) | <0.001^*^ | <0.001^*^ | <0.001^*^ | <0.001^*^ |
| Frontal/Executive | -0.15(0.95) | -0.91(1.08) | -1.14(1.13) | <0.001^*^ | <0.001^*^ | 0.288 | <0.001^*^ |

Bonferroni correction was performed for multiple group comparison. HV = hippocampal volume, K-MMSE = Korean version of mini-mental state examination
