# Supplementary material for: Staging and quantification of florbetaben PET images using machine learning: impact of predicted regional cortical tracer uptake and amyloid stage on clinical outcomes
Source: Eur J Nucl Med Mol Imaging. 2019 Dec 28;47(8):1971–83. doi: 10.1007/s00259-019-04663-3 (PMC7299909; doi:10.1007/s00259-019-04663-3)
Supplement: Supplementary file 3 — (DOCX 97 kb) [file 259_2019_4663_MOESM3_ESM.docx]

**Staging and quantification of florbetaben PET images using machine learning: Impact of predicted regional cortical tracer uptake and amyloid stage on clinical outcomes**

Jun Pyo Kim^1,2,3*^, Jeonghun Kim^4*^, Yeshin Kim^9^, Seung Hwan Moon^10^, Yu Hyun Park^1,2^, Sole Yoo^11^, Hyemin Jang^1,2,3^, Hee Jin Kim^1,2,3^, Duk L. Na^1,2,3,6^, Sang Won Seo^1,2,3,7,8†^, Joon-Kyung Seong^4,5†^

* These authors contributed equally to this work

†These authors contributed equally to this work

^1^Department of Neurology, Samsung Medical Center, Seoul, Korea

^2^Samsung Alzheimer Research Center, Samsung Medical Center, Seoul, Korea

^3^Neuroscience Center, Samsung Medical Center, Seoul, Korea

^4^Department of Bio-convergence Engineering, Korea University, Seoul, Korea

^5^School of Biomedical Engineering, Korea University, Seoul, Korea

^6^Department of Health Sciences and Technology, SAIHST, Sungkyunkwan University, Seoul, Korea

^7^Department of Clinical Research Design & Evaluation, SAIHST, Sungkyunkwan University, Seoul, Korea

^8^Center for Clinical Epidemiology, Samsung Medical Center, Seoul, Korea

^9^Kangwon National University Hospital, Chuncheon, Korea

^10^Department of Nuclear Medicine, Samsung Medical Center, Seoul, Korea

^11^Department of Cognitive Science, Yonsei University, Seoul, Republic of Korea

**Address for correspondence:**

Joon-Kyung Seong, PhD

School of Biomedical Engineering, Korea University

145 Anam-ro, Seongbuk-gu, Seoul, Republic of Korea

Tel: +82-2-3290-5660, Fax: +82-2-921-6434

Email: jkseong@korea.ac.kr

Sang Won Seo, MD PhD

Department of Neurology, Samsung Medical Center, Sungkyunkwan University, School of Medicine,

81 Irwon-ro, Gangnam-gu, Seoul, 06351, South Korea

Tel.: 82-2-3410-1397, Fax: 82-2-3410-0052

Email: [sangwonseo@empal.com](mailto:sangwonseo@empal.com)





Online Resource 3. Diagrams of path analysis.

Standardised coefficients are presented on the path. The models were controlled for age, sex, and education.

*p < 0.05, **p < 0.01, ***p < 0.001

pRCTU = predicted regional cortical tracer uptake
